# Supplementary material for: Identification of photoperiod-induced specific miRNAs in the adrenal glands of Sunite sheep (Ovis aries)
Source: Front Vet Sci. 2022 Jul 22;9:888207. doi: 10.3389/fvets.2022.888207 (PMC9354845; doi:10.3389/fvets.2022.888207)
Supplement: Supplementary file 1 [file Data_Sheet_1.ZIP › Supplementary file/miRDeep2 file.zip!/miRDeep2 file/Novel_Structure/SPLP42A3/Novel_340.pdf]

Provisional ID	:	NC_019460.2_7693
Score total	:	2.3
Score for star read(s)	:	-1.3
Score for read counts	:	0
Score for mfe	:	2
Score for randfold	:	1.6
Score for cons. seed	:	
Total read count	:	817
Mature read count	:	817
Loop read count	:	0
Star read count	:	0

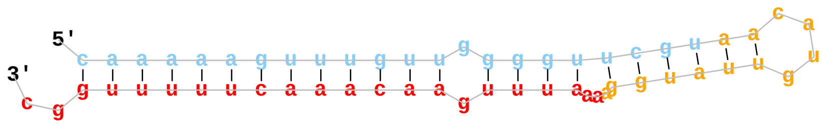

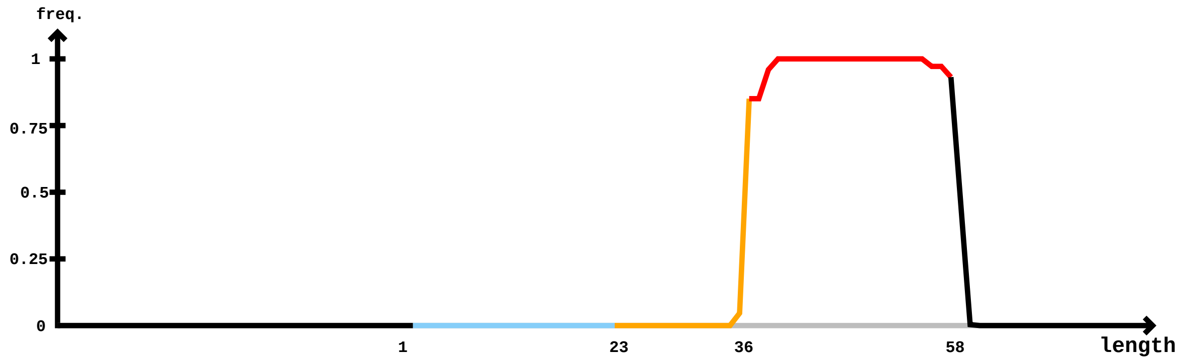

Star Mature

```
5'- accacacucuggaaaucaguaaauauuggguugguCaaaaaguuuguugggguucguaacauguuauggaaaauuugaacaaacuuuuuggccaacccaguauuauaaacca   -3'   exp
    .(((.....))).........(((((((((((((((((((((((((((.((((((((((....))))))...)))).)))))))))))))))))))))))))))........    reads   mm   sample
    .....................................................................aaaauCugaacaaacuuuuu.......................    18      1    seq
    .....................................................................aaaauuugaacaaacuuuuu.......................    1       0    seq
    .....................................................................aaaauCugaacaaacuuuuugg.....................    19      1    seq
    ......................................................................aaauGugaacaaacuuuuu.......................    1       1    seq
    ......................................................................aaauCugaacaaacuuuuu.......................    3       1    seq
    ......................................................................aaauCugaacaaacuuuuugg.....................    14      1    seq
    ......................................................................aaauCugaacaaacuuuuuggc....................    637     1    seq
    ......................................................................aaauuugaacaaacuuuuuggc....................    2       0    seq
    ........................................................................auCugaacaaacuuuuuggc....................    16      1    seq
    ........................................................................auCugaacaaacuuuuuggcc...................    73      1    seq
    .........................................................................uCugaacaaacuuuuuggcc...................    30      1    seq
    .........................................................................uCugaacaaacuuuuuggcca..................    3       1    seq
```
